# Hospital-acquired pneumonia caused by multidrug-resistant *Streptococcus pneumoniae* serotype 15A

**DOI:** 10.1007/s15010-025-02652-3

**Published:** 2025-09-23

**Authors:** Hidemasa Akazawa, Shinnosuke Fukushima, Kenta Nakamoto, Kohei Oguni, Madoka Shimbe, Bin Chang, Yukihiro Akeda, Hideharu Hagiya

**Affiliations:** 1 Department of Infectious Diseases, Okayama University Hospital, 2-5-1 Shikata-cho, Kitaku, Okayama 700-8558 Japan; 2 Department of Bacteriology, Dentistry and Pharmaceutical Sciences, Okayama University Graduate School of Medicine, Okayama, Japan; 3 Department of General Medicine, Okayama University Hospital, Okayama, Japan; 4 Department of Bacteriology I, National Institute of Infectious Diseases, Japan Institute for Health Security, Tokyo, Japan

**Keywords:** Antimicrobial resistance, Multidrug-resistant, Nosocomial infection, Sequence type 63, Serotype 15A, *Streptococcus pneumoniae*

## Abstract

**Background:**

*Streptococcus pneumoniae* remains a common cause of community-acquired pneumonia but is an infrequent pathogen in hospital-acquired pneumonia (HAP). Non-vaccine serotypes of multidrug-resistant (MDR) *S. pneumoniae* strains have been emerging globally, posing an increased risk of nosocomial infection.

**Case:**

A 71 year-old man developed pneumonia on postoperative day 4 following spinal fusion surgery. Despite initial treatment with ampicillin/sulbactam, his condition deteriorated, requiring ICU admission and mechanical ventilation. Microbiological testing confirmed *S. pneumoniae* as a causative pathogen, and ceftriaxone was empirically administered based on the local antibiogram. However, antimicrobial susceptibility testing revealed resistant profiles to penicillin (minimum inhibitory concentration [MIC], 8 µg/mL), ceftriaxone (MIC, 16 µg/mL), meropenem (MIC, 1 µg/mL), macrolides, and clindamycin, while demonstrating susceptibility to levofloxacin and vancomycin. The therapeutic regimen was subsequently adjusted to levofloxacin, resulting in clinical improvement. The isolate was later identified as serotype 15A, sequence type 63 (ST63).

**Conclusion:**

This case highlights that MDR *S. pneumoniae* can cause early-onset HAP and may not be covered by standard empiric therapies, emphasizing the need for careful evaluation of treatment response. Continued surveillance of infections caused by vaccine-escape clones like MDR serotype 15A is essential, given their increasing clinical relevance.

## Introduction

*Streptococcus pneumoniae* is an encapsulated gram-positive diplococcus that commonly causes pneumonia, meningitis, and invasive pneumococcal disease [1]. *S. pneumoniae* accounts for approximately 20–50% of community-acquired bacterial pneumonia cases [2, 3] and 5–9% of hospital-acquired pneumonia (HAP) cases [4, 5]. According to recent surveillance studies, resistance rates based on non-meningeal breakpoints are relatively low, with penicillin G (PCG) resistance reported at 2–4% and ceftriaxone (CTRX) at 2–5%, meropenem (MEPM) at 2.7–33% [6–10].

The global incidence of pneumococcal infections has declined following the introduction of the 13-valent pneumococcal conjugate vaccine (PCV13) [11]. However, an increasing number of infections caused by non-vaccine serotypes [12] and multidrug-resistant (MDR) strains, that is typically defined as resistance to three or more classes of antimicrobial agents [13], have been reported [14]. Among these, serotype 15A has attracted attention as a representative MDR strain not covered by currently available vaccines such as PCV13, PCV15, PCV20, and PPSV23 [14–16]. Here, we report a case of hospital-acquired pneumonia caused by an MDR *S. pneumoniae* serotype 15A.

## Case report

**Fig. 1:**
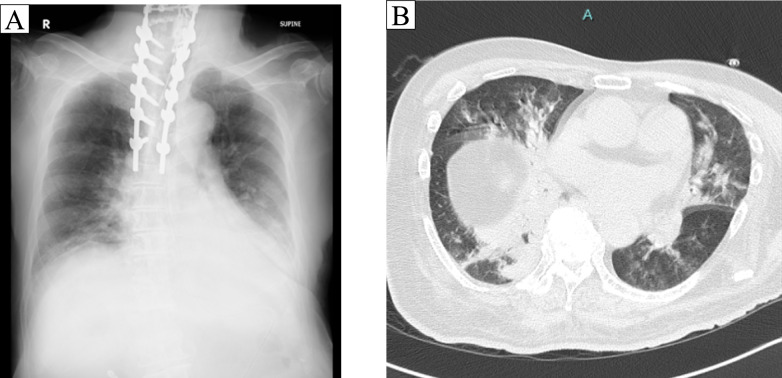
The chest radiograph and chest computed tomography. Both chest radiograph and chest CT revealed consolidation in the right lower lobe

A 71-year-old man underwent simultaneous anterior and posterior spinal fusion surgery for cervical spondylotic myelopathy and was admitted to the intensive care unit (ICU) postoperatively as planned. On postoperative day (POD) 2, the patient was extubated and transferred to the general ward. On POD 4, the patient developed fever, purulent sputum, and decreased oxygen saturation, with the chest X-ray showing right lower lobe consolidation (Fig. 1A). Chest computed tomography revealed consolidation in the dorsal segment of the right lower lobe and mucus accumulation within the bronchi (Fig. 1B). Laboratory tests revealed the following results: white blood cell count, 6730/μL; neutrophils: 86% (segmented neutrophils 78%, band forms 8%); and C-reactive protein, 22.17 mg/dL. Gram staining of a sputum sample classified as P1 by the Miller–Jones classification detected polymicrobial patterns, including encapsulated Gram-positive diplococci. Blood cultures were negative at the onset of clinical deterioration. Considering his advanced age, history of prolonged intubation, and recent cervical surgery, together with the radiographic and microbiological findings, he was diagnosed with aspiration pneumonia and was initially treated with ampicillin/sulbactam.

Later that night, the patient’s respiratory condition deteriorated, accompanied by hypotension, necessitating readmission to the ICU and reintubation. Bronchoscopy revealed obstruction of the left upper and lower lobes with white secretions, and purulent sputum was obtained from the right lower lobe. Bronchial aspirate Gram stain demonstrated Gram-positive diplococci accompanied by phagocytosis, with no other organisms observed. The antimicrobial regimen was empirically switched to the combination of meropenem and vancomycin, considering his deteriorating condition.

On POD 6, the causative pathogen was confirmed to be *S. pneumoniae*, which was identified from the bronchial aspirate two days after the sample collection. Given previous susceptibility data of our hospital indicating a 100% susceptibility rate of *S. pneumoniae* to ceftriaxone, the antimicrobial therapy was de-escalated to ceftriaxone before the susceptibility results became available. Complete antimicrobial susceptibility testing results were issued three days after the sample collection, showing resistance to penicillin G (PCG: MIC, 8 µg/mL), ceftriaxone (CTRX: MIC, ≥ 16 µg/mL), meropenem (MEPM: MIC, 1 µg/mL), azithromycin (AZM: MIC, ≥ 4 µg/mL), and clindamycin (CLDM: MIC, ≥ 8 µg/mL), while susceptibility to vancomycin (VCM: MIC, 0.5 µg/mL) and levofloxacin (LVFX: MIC, 2 µg/mL), as interpreted according to Clinical and Laboratory Standards Institute (CLSI) breakpoints (Table 1). The patient underwent definitive therapy with LVFX, and his subsequent clinical course was favorable.

**Table 1:** Antimicrobial susceptibility of serotype 15A, sequence type 63 *Streptococcus pneumoniae*

		EUCAST		CLSI	
Antimicrobials	MIC (µg/ml)	^a^Breakpoint reference	Susceptibility	^b^Breakpoint reference	Susceptibility
Penicillin G (non-meningitis)	8	S: ≤ 0.06	R	S: ≤ 2	R
Ampicillin	4	S: ≤ 0.5	R	n.d	–
Ampicillin-sulbactam	4	n.d	–	n.d	–
Amoxicillin-clavulanate	4	n.d	–	S: ≤ 2/1	I
Cefotaxime (non-meningitis)	≥ 16	S: ≤ 0.5	R	S: ≤ 1	R
Cefditoren pivoxil	≥ 16	n.d	–	n.d	–
Meropenem	1	S: ≤ 2	S	S: ≤ 0.25	R
Tebipenem	0.12	n.d	–	n.d	–
Erythromycin	≥ 8	S: ≤ 0.25	R	S: ≤ 0.25	R
Clarithromycin	≥ 16	S: ≤ 0.25	R	S: ≤ 0.25	R
Azithromycin	≥ 4	S: ≤ 0.25	R	S: ≤ 0.5	R
Clindamycin	≥ 8	S: ≤ 0.5	R	S: ≤ 0.25	R
Trimethoprim-sulfamethoxazole	1/19	S: ≤ 0.5/9.5	I	S: ≤ 0.5/9.5	I
Levofloxacin	2	S: ≤ 0.001	R	S: ≤ 2	S
Vancomycin	0.5	S: ≤ 2	S	S: ≤ 1	S

*MIC* minimum inhibitory concentration, *R* resistant, *I* intermediate, *S* susceptible, *n.d.* not defined

^a^MIC breakpoint reference EUCAST: European Committee on Antimicrobial Susceptibility Testing v 15.0

^b^MIC breakpoint reference CLSI: M100 (34th edition) by the Clinical and Laboratory Standards Institute

MICs were determined using the Eiken Dry Plate (Eiken Chemical Co., Ltd., Tokyo, Japan), a broth microdilution-based method

The pneumococcal isolate was transferred to the National Institute of Infectious Diseases (Tokyo, Japan), where it was serotyped by a capsule Quellung reaction with serotype-specific rabbit antiserum (Statens Serum Institute, Copenhagen, Denmark). Multilocus sequence typing was performed by sequencing seven housekeeping genes (*aroE, gdh, gki, recP, spi, xpt, ddl*) [17]. Consequently, it was determined to be serotype 15A and sequence type (ST) 63.

## Discussion

We experienced a case of nosocomial pneumonia caused by serotype *S. pneumoniae* 15A-ST63, which exhibits an MDR phenotype. This serotype corresponds to the representative MDR clone reported in the Pneumococcal Molecular Epidemiology Network (PMEN), whose global prevalence has been rising in recent years [18]. Before the introduction of PCVs, only 6.5% of ST63 strains were classified as serotype 15A; however, its proportion soared to 65.4% in the post-PCV era [19]. Importantly, the serotype 15A-ST63 clone has drawn growing attention for its resistance to β-lactam antibiotics commonly used in clinical settings [18, 20, 21]. It is noteworthy that, although the notable serotype has not been included in the existing vaccines, it is finally incorporated into the recently introduced PCV21 [14–16].

The present isolate of *S. pneumoniae* demonstrated a broad spectrum of antimicrobial resistance, including PCG, CTX, MEPM, AZM, and CLDM. The acquisition of broad β-lactam resistance in *S. pneumoniae* is largely attributed to mutations and genetic recombination in penicillin-binding protein (PBP) genes. Although six PBPs (PBP1a, PBP1b, PBP2a, PBP2x, PBP2b, and PBP3) are encoded in the *S. pneumoniae* genome, resistance to β-lactam antibiotics is primarily associated with alterations in PBP1a, PBP2x, and PBP2b [13]. Nakano et al*.* performed whole-genome sequencing of serotype 15A-ST63 isolates with reduced susceptibility to MEPM, reporting that recombination events in the *pbp1a* and *pbp2b* regions were responsible for this phenotype [18]. The *pbp1a* gene identified in these isolates was identical to that of the serotype 19A-ST320 clone, another MDR strain with high-level MEPM resistance that emerged in the United States, and this recombination event has been shown to confer reduced susceptibility to carbapenems [22]. Furthermore, recombination in the *pbp2x* gene, which can be observed in the ST63 lineage, is known to play a critical role in cephalosporin resistance [20]. Macrolide resistance in *S. pneumoniae* is predominantly mediated by two mechanisms: ribosomal target modification via the *erm(B)*-encoded erythromycin ribosomal methylase, and active efflux pump by the major facilitator superfamily transporter encoded by *mefA* or *mefE* [13]. Although molecular testing, such as PCR or whole-genome sequencing, was not performed in this case, the resistance phenotype observed in the present isolate—PCG-resistant, CTRX-resistant, and MEPM-resistant—suggests the presence of broad β-lactam resistance mediated by genetic alterations in *pbp1a* and *pbp2x*. In addition, the concurrent resistance to macrolides and lincosamides implies involvement of the *erm(B)* gene and associated methyltransferases in mediating ribosomal target modification. Recent reports suggest that rapid whole-genome sequencing enables early identification of capsular and serotype profiles [23]. Once extended to resistance gene detection, this strategy may contribute to the timely modification of antimicrobial regimens in high-risk populations.

In general, typical pathogens of HAP include *Pseudomonas aeruginosa, Acinetobacter baumannii* complex, and *Staphylococcus aureus*, while *S. pneumoniae* is a relatively uncommon cause [5]. However, *S. pneumoniae* can be a causative organism in early-onset HAP (defined as onset within 4–7 days of hospitalization), accounting for approximately 9% of cases [4]. A nosocomial cluster caused by MDR *S. pneumoniae* serotype 15A was reported in the Netherlands [24], highlighting the potential risk of nosocomial transmission of MDR *S. pneumoniae*. In our case, although active contact tracing was not conducted, no additional isolates were identified thereafter. However, this case reminded us of the importance of maintaining strict infection control vigilance when unusual or multidrug-resistant pneumococci are detected.

Collectively, we presented an early-onset HAP case of *S. pneumoniae* serotype 15A-ST63*.* This case underscores the need to consider MDR pneumococcal infection in hospitalized patients, highlighting both the potential necessity for early adjustment of antimicrobial coverage in high-risk individuals and the importance of carefully evaluating treatment response. Furthermore, the potential risk of nosocomial transmission of MDR *S. pneumoniae* should be emphasized from both clinical and infection control perspectives. Finally, prospective epidemiological investigations are warranted to delineate patient populations for whom empirical MDR pneumococcal coverage should be considered.

## Data Availability

No datasets were generated or analysed during the current study.
